# Current Status of Dental Examinations During Pregnancy Term in Matsudo City, Japan

**DOI:** 10.7759/cureus.80533

**Published:** 2025-03-13

**Authors:** Keiichi Kumasawa, Chieko Taguchi, Itaru Suzuki, Kazumune Arikawa

**Affiliations:** 1 Obstetrics and Gynecology, University of Tokyo, Tokyo, JPN; 2 Hygiene, Nihon University School of Dentistry at Matsudo, Matsudo, JPN

**Keywords:** female, japan, pregnancy complications, pregnant women, toothbrushing

## Abstract

Aim: Poor oral hygiene during pregnancy is associated with pre-term birth, low birth weight, and pregnancy-induced hypertension, suggesting its significant effects on both maternal and fetal health. However, global guidelines indicate that oral care during pregnancy remains an underdeveloped field, and no large-scale reports on dental studies conducted during pregnancy exist in Japan. Therefore, we assessed the key factors that contribute to maintaining oral health during pregnancy in a cohort of Japanese women.

Methods: We conducted dental examinations on 907 pregnant women in Matsudo City, Japan, and investigated the key factors for maintaining 379 women (41.7%) had untreated cavities. An increase in the frequency of toothbrushing was associated with a higher number of treated teeth (r=0.315, p < 0.001). However, periodontal disease-related factors such as gingival bleeding (71.2%) and the condition of periodontal pockets showed little correlation with brushing frequency(r=-0.139, p > 0.05 for untreated teeth and brushing frequency).

Conclusions: While brushing teeth three times a day, including during pregnancy, is ideal, attention should also be directed to other aspects, such as brushing technique and thoroughness, which may require further consideration. This report serves as a foundation for obstetricians and dentists to recognize the importance of oral hygiene management during pregnancy and advocate for dental checkups during pregnancy, providing valuable opportunities to increase awareness regarding this issue.

## Introduction

Oral health conditions during pregnancy have been correlated with various pregnancy complications, including pre-term birth, hypertensive disorders of pregnancy, and low birth weight [1-5]. Maintaining optimal oral health during pregnancy plays a critical role in ensuring the well-being of both the mother and fetus. However, hormonal fluctuations and dietary changes during pregnancy can affect the oral environment considerably. For instance, pregnancy-related symptoms, such as nausea and vomiting as well as the mechanical effects of uterine expansion on the stomach, may decrease food intake per meal [6]. Additionally, the elevated risk of gastroesophageal reflux due to lower esophageal sphincter relaxation can increase oral acidity and potentially alter the oral microbiota [7]. Such changes may hinder pregnant women from maintaining their pre-pregnancy oral hygiene habits such as regular toothbrushing and the use of interdental brushes.

Global guidelines on oral care during pregnancy currently recognize this field as underdeveloped, with few comprehensive studies conducted in Japan on the state of dental health in pregnant women [8]. Despite advancements in the management of complicated pregnancies and development of advanced reproductive technologies in Japan, studies on the relationship between oral health and pregnancy remain limited.Therefore, in this study, we investigated the key factors, such as frequency of toothbrushing, that contribute to maintaining oral health during pregnancy in a cohort of Japanese women, with the aim of providing essential insights into the unique oral health risks and preventive strategies for pregnant women.

## Materials and methods

Study participants and data

In this cross-sectional study, we included 907 pregnant women from Matsudo City, Chiba Prefecture, Japan, who voluntarily underwent prenatal dental checkups between April 1, 2021, and March 31, 2022, as part of Matsudo City’s prenatal examination subsidy program. The participants’ age was based on the Japanese academic year, specifically, their age as of March 31, 2022.

Data on gestational age (categorized as up to 15 weeks, 16-27 weeks, and 28-39 weeks) and oral health habits, including the frequency of toothbrushing (0, 1, 2, or 3+ times per day) and interdental brush/floss usage (0 times, 1-3 times per month, once or more per week, or daily), were collected through self-reported questionnaires administered to pregnant women and from maternal and child health handbooks. Information on dental checkup frequency within the previous year (yes or no) and smoking history (never smoked, past smoker, or current smoker) was also obtained. Dental health indicators, such as the number of teeth, treated and untreated teeth, current teeth, gingival bleeding status (- or +), periodontal pocket status (healthy, shallow, or deep), oral hygiene condition, and tartar accumulation (no, minor, more than moderately), were evaluated and recorded by dentists.

Ethical approval

This study was conducted in compliance with the ethical guidelines outlined in the Declaration of Helsinki, and the protocol was approved by the Ethics Committee of Nihon University School of Dentistry at Matsudo (Approval No.: EC22-023). The requirement for informed consent was waived because personal data were fully anonymized before the analysis.

Statistical analysis

Statistical analyses were performed using IBM SPSS Statistics, version 29 (IBM Corp., Armonk, USA). Chi-square tests, Fisher’s exact tests, and Spearman’s rank correlation coefficient analyses were conducted to evaluate associations across variables. We have specified the significance level as p < 0.05 throughout the article.

Regarding missing data, cases with missing values were excluded from the analysis using a complete case approach.

## Results

Participant characteristics and dental habits

Age Distribution and Gestational Weeks at the Time of Examination

The age distribution of the 907 pregnant women who underwent dental checkups in Matsudo City during the 2021 fiscal year was as follows: 279 women in their teens and 20s (30.8%), 581 in their 30s (64.1%), and 47 in their 40s (5.2%). In terms of gestational weeks at the time of examination, 55 women were in the first 15 weeks of pregnancy (6.1%), 658 in weeks 16-27 (72.5%), and 191 in weeks 28-39 (21.1%). The distribution of gestational weeks by age is shown in Table 1. Women in the two younger groups underwent a high frequency of checkups by the 27th week (82.4, and 77.8%, respectively) compared to those in their 40s (66.0%).

**Table 1:** Gestational week at dental examination Statistical analysis was performed using the Chi-square test. Fisher’s exact test was used when the expected cell count was less than 5. Groups within the same column that share the same subscripted letter ('a' or 'b') do not significantly differ from each other at the 0.05 level, based on pairwise comparisons. Groups with different letters are significantly different.

			Gestational week at dental examination (week)					
				≤15	16-27	28-39	Total	
Age	≦29	N		21 _b_	209 _a, b_	47 _a_	279	
		%		7.5%	74.9%	16.8%	100.0%	
	30-39	N		31_a_	421_a_	128_a_	581	
		%		5.3%	72.5%	22.0%	100.0%	
	40-49	N		3 _a, b_	28 _b_	16 _a_	47	
		%		6.4%	59.6%	34.0%	100.0%	
Total		N		55	658	191	907	
		%		6.1%	72.5%	21.1%	100.0%	

Dental Checkups Within the Last Year

Of the 907 women, 344 (37.9%) underwent a dental checkup within the previous year, 556 (61.3%) did not, and 7 (0.8%) did not respond to this item (Table 2). There were no significant differences in checkup frequency among the age groups.

**Table 2:** Dental visits within one year Statistical analysis was performed using the Chi-square test. Fisher’s exact test was used when the expected cell count was less than 5. Groups within the same column that share the same subscripted letter ('a' or 'b') do not significantly differ from each other at the 0.05 level, based on pairwise comparisons. Groups with different letters are significantly different.

		Age						Total	
		≤29		30-39		40-49			
		N	%	N	%	N	%	N	%
Dental examination within a year	N/A	2_a_	0.7%	5_a_	0.9%	0_a_	0.0%	7	0.8%
	Yes	92_a_	33.0%	238_b_	41.0%	14_a, b_	29.8%	344	37.9%
	No	185_a_	66.3%	338_b_	58.2%	33_a, b_	70.2%	556	61.3%
Total		279	100.0%	581	100.0%	47	100.0%	907	100.0%

Toothbrushing Frequency

The percentage of women who brushed their teeth three or more times per day was higher among those in their 30s (39.8%) and 40s (46.8%) than among those in their 20s (29.0%; p=0.023) (Table 3).

**Table 3:** Relationship between age and the number of times a day one brushes one's teeth Statistical analysis was performed using the Chi-square test. Fisher’s exact test was used when the expected cell count was less than 5. Groups within the same column that share the same subscripted letter ('a' or 'b') do not significantly differ from each other at the 0.05 level, based on pairwise comparisons. Groups with different letters are significantly different. One participant in the 30-39 age group was marked as N/A.

		Age						Total		
		≤29		30-39		40-49				
		N	%	N	%	N	%	N	%	
Number of times per day to brush teeth	0	1_a_	0.4%	0_a_	0.0%	0_a_	0.0%	1	0.1%	
	1	28_a_	10.0%	44_a_	7.6%	5_a_	10.6%	77	8.5%	
	2	169_a_	60.6%	305_b_	52.6%	20_b_	42.6%	494	54.5%	
	3	81_a_	29.0%	231_b_	39.8%	22_b_	46.8%	334	36.9%	
Total		279	100.0%	580	100.0%	47	100.0%	906	100.0%	

Use of Interdental Brushes and Floss by Age Group

The rate of daily flossing or interdental brush use was low across all the age groups: 9.3% among women in their teens and 20s, 18.9% among those in their 30s, and 21.3% among those in their 40s. However, the rate was lower among women in their 20s than among those in other age groups (Table 4).

**Table 4:** Relationship between age and the number of interdental brushes/flosses per day Statistical analysis was performed using the Chi-square test. Fisher’s exact test was used when the expected cell count was less than 5. Groups within the same column that share the same subscripted letter ('a' or 'b') do not significantly differ from each other at the 0.05 level, based on pairwise comparisons. Groups with different letters are significantly different.

		Age						Total	
		≤29		30-39		40-49			
		N	%	N	%	N	%	N	%
Floss, interdental brush	No use	134_a_	48.0%	189_b_	32.5%	15_b_	31.9%	338	37.3%
	1-3 times a month	64_a_	22.9%	134_a_	23.1%	7_a_	14.9%	205	22.6%
	More than once a week	53_a_	19.0%	146_b_	25.1%	15_b_	31.9%	214	23.6%
	Every day	26_a_	9.3%	110_b_	18.9%	10_b_	21.3%	146	16.1%
Total		279	100.0%	581	100.0%	47	100.0%	907	100.0%

Chewing Habits

Among all the participants, 847 (93.4%) reported chewing their food thoroughly, whereas 60 (6.6%) reported not chewing it thoroughly. According to age, the rates were 93.5% among women in their teens and 20s, 93.5% among those in their 30s, and 91.5% among those in their 40s, with no significant differences between age groups (Table 5). According to gestational age, the rate of not chewing thoroughly was 10.9%, 9.4%, and 5.4% among those at gestational weeks 0-15, 28-39, and 16-27, respectively (Table 6).

**Table 5:** Age-related differences in chewing habits during pregnancy Statistical analysis was performed using the Chi-square test. Fisher’s exact test was used when the expected cell count was less than 5. The subscripted lowercase letter 'a' signifies that the values within the same column are not significantly different from each other at the 0.05 level, as determined by pairwise comparisons.

		Age						Total		
		≤29		30-39		40-49				
		N	%	N	%	N	%	N	%	
Chew well or not	No	18_a_	6.5%	38_a_	6.5%	4_a_	8.5%	60	6.6%	
	Yes	261_a_	93.5%	543_a_	93.5%	43_a_	91.5%	847	93.4%	
Total		279	100.0%	581	100.0%	47	100.0%	907	100.0%	

**Table 6:** Chewing habits by gestational age during pregnancy Statistical analysis was performed using the Chi-square test. Fisher’s exact test was used when the expected cell count was less than 5. The subscripted lowercase letter 'a' signifies that the values within the same column are not significantly different from each other at the 0.05 level, as determined by pairwise comparisons.

		Gestational age						Total	
		0-15		16-27		28-39			
		N	%	N	%	N	%	N	%
Chew well or not	No	6_a_	10.9%	36	5.5%	18_a_	9.4%	60	6.6%
	Yes	49_a_	89.1%	620	94.2%	173_a_	90.6%	842	93.2%
	N/A	0	0	5	0.6	0	0	5	0.3
Total		55	100.0%	661	100.0%	191	100.0%	907	100.0%

Smoking Habits

A trend was observed in which an older age was associated with a higher rate of past smoking (Table 7). At the time of the dental check-up, only one woman in the teens to 20s age group reported to be currently smoking.

**Table 7:** History of smoking Statistical analysis was performed using the Chi-square test. Fisher’s exact test was used when the expected cell count was less than 5. Groups within the same column that share the same subscripted letter ('a' or 'b') do not significantly differ from each other at the 0.05 level, based on pairwise comparisons. Groups with different letters are significantly different.

		Age						Total	
		≤29		30-39		40-49			
		N	%	N	%	N	%	N	%
History of smoking	Never	236_a_	84.6%	476_a_	81.9%	31_b_	66.0%	743	81.9%
	Previous	40_a_	14.3%	104_a_	17.9%	16_b_	34.0%	160	17.6%
	Current smoker	1_a_	0.4%	0_a_	0.0%	0_a_	0.0%	1	0.1%
Total		279	100.0%	581	100.0%	47	100.0%	907	100.0%

Dental examination results

Number of Current, Healthy, Treated, and Untreated Teeth

The average number of remaining teeth was 28.6±1.7 among women in their teens and 20s, 28.4±1.7 among those in their 30s, and 28.1±1.4 among those in their 40s (Table 8). In terms of healthy teeth, the average number was 21.7±5.5 among women in their teens and 20s, 19.4±5.5 among those in their 30s, and 15.4±6.1 among those in their 40s (Table 8). The average number of treated teeth was 5.4±4.8 among women in their teens and 20s, 7.7±5.1 among those in their 30s, and 11.4±6.1 among those in their 40s (Table 8). The average number of untreated teeth was 1.5±2.7 among women in their teens and 20s, 1.3±2.2 among those in their 30s, and 1.3±2.0 among those in their 40s (Table 8). No significant correlation was found between the number of healthy teeth and the frequency of toothbrushing or the use of floss and interdental brushes (Table 9). A very weak negative correlation was found between the number of untreated teeth and toothbrushing frequency (r=-0.139) and the use of interdental brushes and floss (r=-0.159) (Table 10). No significant correlation was found between having undergone a dental check-up in the past year and the number of untreated teeth.

**Table 8:** Age distribution of healthy, untreated, treated, and present teeth during pregnancy Age-related trends in the number of healthy, untreated, treated, and present teeth. The table demonstrates a reasonable pattern of changes in these dental categories with advancing age, reflecting the impact of aging and dental treatments on oral health.

	Age					
	≤29		30-39		40-49	
	Average	SD	Average	SD	Average	SD
Number of healthy teeth	21.7	5.5	19.4	5.5	15.4	6.1
Number of teeth untreated	1.5	2.7	1.3	2.2	1.3	2.0
Number of teeth treated	5.4	4.8	7.7	5.1	11.4	6.1
Number of current teeth	28.6	1.7	28.4	1.7	28.1	1.4
N	279		581		47	

**Table 9:** Relationship between oral hygiene practices and dental examination findings during pregnancy Spearman's rank correlation coefficient was used to analyze the relationships between variables. Asterisks indicate statistical significance. *: p < 0.05, **: p < 0.01 PCC: Pearson's correlation coefficient; SP: Significant probability (both sides); N/A: Not available

		Age	Dental exam within a year	Toothbrushes per day	Floss, interdental brush	History of smoking	Present number of teeth	Number of healthy teeth	Number of teeth treated	Number of untreated teeth	Gingival bleeding	Perio-dontal pocket status	Tartar	Oral cleaning condition	
Age	PCC	1	0.042	.103^**^	.088^**^	-0.030	-0.011	-0.024	0.041	-0.040	-0.039	-0.060	-.067^*^	0.058	
	SP		0.211	0.002	0.008	0.374	0.740	0.472	0.219	0.224	0.236	0.073	0.043	0.080	
	N/A	0	7	1	4	3	0	0	0	0	0	0	0	0	
	N	907	900	906	903	904	907	907	907	907	907	907	907	907	
Dental exam within a year	PCC	0.042	1	0.064	-0.028	-0.020	-0.014	0.028	-0.035	-0.001	-0.028	-0.005	0.024	-0.018	
	SP	0.211		0.056	0.407	0.548	0.671	0.409	0.299	0.971	0.395	0.877	0.471	0.599	
	N/A	7	7	8	11	10	7	7	7	7	7	7	7	7	
	N	900	900	899	896	897	900	900	900	900	900	900	900	900	
Toothbrushes per day	PCC	.103^**^	0.064	1	0.036	-.091^**^	-0.006	0.044	-0.007	-.096^**^	-0.051	-.073^*^	-.109^**^	.091^**^	
	SP	0.002	0.056		0.283	0.006	0.848	0.183	0.831	0.004	0.126	0.028	0.001	0.006	
		1	8	1	5	4	1	1	1	1	1	1	1	1	
	N	906	899	906	902	903	906	906	906	906	906	906	906	906	
Floss, interdental brush	PCC	.088^**^	-0.028	0.036	1	-0.033	-.109^**^	-.114^**^	.124^**^	-.074^*^	-0.053	-0.046	-.127^**^	.160^**^	
	SP	0.008	0.407	0.283		0.319	0.001	0.001	0.000	0.026	0.111	0.167	0.000	0.000	
	N	903	896	902	903	900	903	903	903	903	903	903	903	903	
History of smoking	PCC	-0.030	-0.020	-.091^**^	-0.033	1	-0.032	-0.051	0.012	.074^*^	.073^*^	.103^**^	.080^*^	-0.049	
	SP	0.374	0.548	0.006	0.319		0.331	0.125	0.709	0.027	0.027	0.002	0.016	0.145	
	N/A	3	10	4	7	3	3	3	3	3	3	3	3	3	
	N	904	897	903	900	904	904	904	904	904	904	904	904	904	
Present number of teeth	PCC	-0.011	-0.014	-0.006	-.109^**^	-0.032	1	.293^**^	-.074^*^	.166^**^	-0.002	.093^**^	.137^**^	-.094^**^	
	SP	0.740	0.671	0.848	0.001	0.331		0.000	0.026	0.000	0.949	0.005	0.000	0.005	
	N/A	0	7	1	4	3	0	0	0	0	0	0	0	0	
	N	907	900	906	903	904	907	907	907	907	907	907	907	907	
Number of healthy teeth	PCC	-0.024	0.028	0.044	-.114^**^	-0.051	.293^**^	1	-.886^**^	-.262^**^	-.065^*^	-0.050	.071^*^	0.058	
	SP	0.472	0.409	0.183	0.001	0.125	0.000		0.000	0.000	0.049	0.135	0.032	0.081	
	N/A	0	7	1	4	3	0	0	0	0	0	0	0	0	
	N	907	900	906	903	904	907	907	907	907	907	907	907	907	
Number of teeth treated	PCC	0.041	-0.035	-0.007	.124^**^	0.012	-.074^*^	-.886^**^	1	-.110^**^	0.027	-0.003	-.113^**^	-0.006	
	SP	0.219	0.299	0.831	0.000	0.709	0.026	0.000		0.001	0.422	0.937	0.001	0.857	
	N/A	0	7	1	4	3	0	0	0	0	0	0	0	0	
	N	907	900	906	903	904	907	907	907	907	907	907	907	907	
Number of untreated teeth	PCC	-0.040	-0.001	-.096^**^	-.074^*^	.074^*^	.166^**^	-.262^**^	-.110^**^	1	.098^**^	.193^**^	.174^**^	-.195^**^	
	SP	0.224	0.971	0.004	0.026	0.027	0.000	0.000	0.001		0.003	0.000	0.000	0.000	
	N/A	0	7	1	4	3	0	0	0	0	0	0	0	0	
	N	907	900	906	903	904	907	907	907	907	907	907	907	907	
Gingival bleeding	PCC	-0.039	-0.028	-0.051	-0.053	.073^*^	-0.002	-.065^*^	0.027	.098^**^	1	.326^**^	.286^**^	-.375^**^	
	SP	0.236	0.395	0.126	0.111	0.027	0.949	0.049	0.422	0.003		0.000	0.000	0.000	
	N/A	0	7	1	4	3	0	0	0	0	0	0	0		
	N	907	900	906	903	904	907	907	907	907	907	907	907	907	
Periodontal pocket status	PCC	-0.060	-0.005	-.073^*^	-0.046	.103^**^	.093^**^	-0.050	-0.003	.193^**^	.326^**^	1	.306^**^	-.323^**^	
	SP	0.073	0.877	0.028	0.167	0.002	0.005	0.135	0.937	0.000	0.000		0.000	0.000	
	N/A	0	7	1	4	3	0	0	0	0	0	0	0		
	N	907	900	906	903	904	907	907	907	907	907	907	907	907	
Tartar	PCC	-.067^*^	0.024	-.109^**^	-.127^**^	.080^*^	.137^**^	.071^*^	-.113^**^	.174^**^	.286^**^	.306^**^	1	-.518^**^	
	SP	0.043	0.471	0.001	0.000	0.016	0.000	0.032	0.001	0.000	0.000	0.000		0.000	
	N/A	0	7	1	4	3	0	0	0	0	0	0	0		
	N	907	900	906	903	904	907	907	907	907	907	907	907	907	
Oral cleaning condition	PCC	0.058	-0.018	.091^**^	.160^**^	-0.049	-.094^**^	0.058	-0.006	-.195^**^	-.375^**^	-.323^**^	-.518^**^	1	
	SP	0.080	0.599	0.006	0.000	0.145	0.005	0.081	0.857	0.000	0.000	0.000	0.000		
	N/A	0	7	1	4	3	0	0	0	0	0	0	0	0	
	N	907	900	906	903	904	907	907	907	907	907	907	907	907	

**Table 10:** Association of toothbrushing and interdental cleaning frequency with the number of untreated teeth The table illustrates the correlation between age and daily toothbrushing frequency. A negative correlation was observed. Statistical analysis was performed using Spearman’s rank correlation coefficient.

Number of untreated teeth	1	2	3	4	5	6	7	8	9	10
Correlation coefficient	-0.228	-0.25	-0.266	-0.278	-0.287	-0.29	-0.291	-0.293	-0.294	-0.297

Gingival Bleeding

Gingival bleeding occurred in 646 of the 907 participants (71.2%): 71.3% of those in their teens and 20s, 70.2% of those in their 30s, and 83.0% of those in their 40s, with no significant differences between age groups (Table 11). The correlation coefficients with other factors were 0.032 for periodontal pocket status, -0.381 for oral cleanliness, and 0.290 for calculus (Table 9).

**Table 11:** Age-related trends in gingival bleeding during pregnancy Statistical analysis was performed using the Chi-square test. Fisher’s exact test was used when the expected cell count was less than 5. The subscripted lowercase letter 'a' signifies that the values within the same column are not significantly different from each other at the 0.05 level, as determined by pairwise comparisons.

		Age						Total	
		≤29		30-39		40-49			
		N	%	N	%	N	%	N	%
Gingival bleeding	－	80_a_	28.7%	173_a_	29.8%	8_a_	17.0%	261	28.8%
	＋	199_a_	71.3%	408_a_	70.2%	39_a_	83.0%	646	71.2%
Total		279	100.0%	581	100.0%	47	100.0%	907	100.0%

Periodontal Pocket Status

The periodontal pocket status was classified as healthy in 400 women (44.1%) and shallow in 395 women (43.6%) (Table 12). The correlation coefficients were -0.326 for oral cleanliness and -0.517 for calculus. There was weak correlation between periodontal pocket status and toothbrushing or interdental brush use (Table 9).

**Table 12:** Periodontal pocket status during pregnancy Statistical analysis was performed using the Chi-square test. Fisher’s exact test was used when the expected cell count was less than 5. The subscripted lowercase letter 'a' signifies that the values within the same column are not significantly different from each other at the 0.05 level, as determined by pairwise comparisons.

		Age						Total	
		≤29		30-39		40-49			
		N	%	N	%	N	%	N	%
Periodontal pocket	Healthy	115_a_	41.2%	264_a_	45.4%	21_a_	44.7%	400	44.1%
	Shallow pocket	120_a_	43.0%	252_a_	43.4%	23_a_	48.9%	395	43.6%
	Deep pocket	44_a_	15.8%	65_a_	11.2%	3_a_	6.4%	112	12.3%
Total		279	100.0%	581	100.0%	47	100.0%	907	100.0%

Oral Cleanliness

Oral cleanliness was classified as poor in 126 women (13.9%), fair in 568 women (62.6%), and good in 213 women (23.5%). A moderate negative correlation was found between oral cleanliness and gingival bleeding (-0.381) and periodontal pocket status (-0.326); however, the correlation with toothbrushing or interdental brush use was weak (Table 9).

Tartar

In total, 185 women (20.4%) had no tartar, 600 (66.2%) had mild tartar, and 122 (13.5%) had moderate or severe tartar (Table 13). A strong negative correlation was found with oral cleanliness (-0.517) and a moderate negative correlation with periodontal pocket status (-0.313) (Table 9).

**Table 13:** Tartar adherence status during pregnancy Statistical analysis was performed using the Chi-square test. Fisher’s exact test was used when the expected cell count was less than 5. Groups within the same column that share the same subscripted letter ('a' or 'b') do not significantly differ from each other at the 0.05 level, based on pairwise comparisons. Groups with different letters are significantly different.

		Age						Total	
		≤29		30-39		40-49			
		N	%	N	%	N	%	N	%
Tartar	No	57_a_	20.4%	115_a_	19.8%	13_a_	27.7%	185	20.4%
	Minor	172_a_	61.6%	397_a_	68.3%	31_a_	66.0%	600	66.2%
	More than moderately	50_a_	17.9%	69_b_	11.9%	3_b_	6.4%	122	13.5%
Total		279	100.0%	581	100.0%	47	100.0%	907	100.0%

## Discussion

Significance of this study

Given the reported associations between the oral health of pregnant women and various perinatal complications, the American Academy of Periodontology recommends incorporating periodontal disease management into pre-pregnancy care programs [9-12]. Improving oral health may enhance pregnancy outcomes and ensure maternal and fetal safety. However, in Japan, the current state of oral health in terms of dental caries and periodontal health among pregnant women is unclear.

During pregnancy, factors such as hormonal changes, nausea, hyperemesis, and lower esophageal sphincter relaxation may impair normal fluid intake and food consumption, complicating the maintenance of oral hygiene practices such as toothbrushing and flossing. Although various studies have reported on changes in the oral microbiome during pregnancy, large-scale studies on dental diseases, such as caries, and periodontal conditions are lacking in Japan. Therefore, the present study holds substantial significance as it provides an assessment of dental practices and the oral health status of pregnant Japanese women. This study does not comprehensively cover the dental visit history of all pregnant women in Matsudo City. For example, it is reasonable to assume that pregnant women who had undergone dental treatment for cavities before or during pregnancy did not undergo the dental checkup conducted in this study. Moreover, many women’s morning sickness or hyperemesis symptoms tend to subside after 14 weeks of gestation, although some women experience persistent symptoms. Those without noticeable symptoms of caries or gingival bleeding due to periodontal disease may be less motivated to undergo dental checkups. Some pregnant women may also choose to avoid medical procedures unrelated to pregnancy and childbirth. Furthermore, women who do not routinely undergo dental checkups or experience a lack awareness of their benefits may be less likely to seek dental care during pregnancy. In Japan, tracking pregnancy-specific migration patterns, such as women opting for hometown deliveries or those receiving prenatal care elsewhere but returning to Matsudo City for delivery, is challenging. However, our report covered approximately 30% of the annual deliveries in Matsudo City in 2021 (3,084 births) and 2022 (2,911 births). Additionally, the average income in Matsudo City was 3.66 million yen in 2022, comparable to Japan’s national average of 3.61 million yen, indicating an average economic profile. This represents a meaningful reflection of Japan's socioeconomic background, though regional biases cannot be entirely excluded.

*Study Design and* *Periods of Evaluation*

In the present study, the participants were divided by gestational week (0-15, 16-27, and 28-39 weeks) rather than by trimester, as the early weeks, particularly up to 15 weeks, are typically affected by morning sickness, which can substantially impact oral health and dental checkup attendance [13]. We observed a lower rate of attendance (6%) among women at up to 15 weeks of pregnancy, compared to 72.5% among those at 16-27 weeks and 21.1% among those at 28-39 weeks. Additionally, many working women take maternity leave at approximately 34 weeks of pregnancy, which may have affected their attendance to dental checkups, especially owing to concerns about supine hypotension syndrome during dental visits and the increased likelihood of food cravings in later pregnancy. Interestingly, the results suggest that women aged 40 years and above may be more likely to attend dental checkups at 28 weeks of gestation or later compared to younger age groups (34.0% vs. 17.0% and 22.1%, p=0.057). However, it is possible that women in their 40s, often in socially responsible or senior positions, may find it more challenging to attend dental checkups immediately before maternity leave begins, approximately at 34 weeks. Their professional responsibilities and potential reluctance to step away from work duties at critical times could lead to lower attendance at this stage of pregnancy. These findings suggest that promoting dental checkups during mid-pregnancy, rather than in the early or late stages, may increase attendance rates among pregnant women.

Annual Dental Checkup Rate

Among the 907 women who underwent dental checkups, 344 underwent checkups within the previous year, while 577 (63.6%) did not. The rate of women who did not undergo a checkup in the previous year in our study was slightly higher than the national rate of 28.6-50.9% among women of comparable age groups [14]. Pregnancy may provide an opportunity for women who do not regularly attend dental checkups to receive care, which would contribute positively to their lifelong oral health. Continued education encouraging postpartum dental checkups could further strengthen these benefits.

Frequency of Toothbrushing

Women in their 30s and 40s reported brushing their teeth three or more times per day, at higher rates than women in their 20s (39.8% and 46.8% vs. 29.0%, p=0.023). This finding is consistent with the 2022 National Survey of Dental Diseases, which reported that women in their 40s brush their teeth more frequently than younger women [14]. The higher brushing frequency observed among women in their 30s in our study may reflect increased health awareness among pregnant women, likely influenced by educational efforts from obstetricians and dentists. Two potential explanations for these age-related differences are an increased awareness of oral health with age and differing levels of health education across age groups, with younger women potentially being less attentive to oral hygiene. Continued education aimed at enhancing awareness of oral health could prove beneficial.

Use of Interdental Brushes and Floss

The use of interdental brushes and floss varied with age, with a lower usage rate among women in their 20s. Comparatively, data from the 2022 National Survey of Dental Diseases revealed that 40-70% of women use these tools regularly, with a higher usage rate among women in their 30s and 40s [14].

Other Dietary and Lifestyle Factors

Approximately 93.2% of the women included in the present study reported chewing their food thoroughly, while the remaining 6.6% reported chewing their food insufficiently. This pattern was consistent across age groups but showed some fluctuations by gestational week. Notably, women in the early weeks of pregnancy reported more difficulty in chewing, likely due to nausea. Nonetheless, the high rate of thorough chewing found across all stages of pregnancy is promising.

Smoking 

We found that the rate of past smoking increased with age, consistent with broader trends showing decreased smoking rates in younger generations. Only one participant was a current smoker at the time of the dental checkup, a positive finding given the well-established adverse effects of smoking during pregnancy, including increased risks of pre-term birth and low birth weight [15,16].

Pregnancy examination results

Number of Current Teeth

The number of current teeth was 28.6±1.7 among women ≤29, 28.4±1.7 among those in their 30s, and 28.1±1.4 among those in their 40s. Statistically, the number of teeth decreased significantly between the group ≤29 and the group in their 30s (p=0.023); however, this was not considered a clinically significant difference.

Number of Healthy Teeth

We found that younger pregnant women had more healthy teeth than did older pregnant women, which is expected considering that the number of healthy teeth decreases with age. Our findings are consistent with those of the 2022 National Survey of Dental Diseases (≤ 29 : 21.7 vs. 22.2-27.3; 30s: 19.4 vs. 18.8-23.12; 40s: 15.4 vs. 14.9-19.6), revealing low numbers of healthy teeth in older groups [12]. This is because the number of dental checkups is low during pregnancy. Although it is desirable to brush one's teeth at least three times per day, we found that the number of times pregnant women brushed their teeth did not correlate with the number of healthy teeth [17,18]. This finding may suggest that the number of healthy teeth did not suddenly decrease during pregnancy, but may instead be a result of toothbrushing habits established prior to pregnancy. Therefore, pregnant women may maintain a healthy number of teeth even if they do not brush their teeth or use interdental brushes to the greatest extent during pregnancy, including during periods of morning sickness and malaise. It is desirable to continue to build evidence and establish guidelines for dental care during pregnancy. Additionally, the paradoxical finding regarding the number of healthy teeth being higher among pregnant women who underwent checkups in the previous year than among those who did not should be noted. In this regard, a reduced frequency of regular checkups due to habits such as proper toothbrushing and the use of interdental brushes and floss may lead to fewer detected caries, potentially decreasing the perceived need for dental visits.

Number of Treated Teeth

We found that the increased number of treated teeth with age was inversely correlated with the number of healthy teeth (r=-0.881). Considering that the number of treated teeth in the corresponding age groups in the 2022 National Survey of Dental Diseases ranged from 0.5 to 4.4 in individuals in their teens and 20s (our study: 5.40±4.77), 4.4 to 8.1 in those in their 30s (our study: 7.70±5.06), and 7.4 to 11.8 in those in their early 40s (our study: 11.4±6.1), our findings revealed high numbers among our sample of pregnant women. Similar to the discussion on the number of healthy teeth, this finding may be due to pregnant women who do not usually receive dental checkups having received such checkups owing to their incorporation in the Maternal and Child Health Handbook.

Number of Untreated Teeth

A minor inverse correlation was found between the number of times the participants brushed their teeth and whether or not they had at least one untreated tooth (r=-0.184). Furthermore, the correlation coefficient between the frequency of toothbrushing and the number of untreated teeth was -0.258; as the number of untreated teeth increased from 3 to 4, 5, and 6, the correlation coefficients increased from -0.311 to -0.358, -0.391, and -0.406, respectively, indicating that the frequency of toothbrushing is strongly correlated with the number of untreated teeth. Although it was difficult to continuously evaluate the correlation between the frequency of toothbrushing before and after pregnancy in this study, the general frequency (before and during pregnancy) should be three times per day as far as possible. However, no similar relationship was found between the frequency of toothbrushing and interdental brushing and flossing. In contrast, there was almost no correlation between the presence or absence of a dental check-up within the previous year and the number of untreated teeth. Not only was the correlation coefficient -0.065 for the question on the “presence of one or more untreated teeth”, but the absolute value of the correlation coefficient remained small as the number of untreated teeth increased. We only gathered data on dental examinations within the previous year, and it may be necessary to examine the relationship between the number of untreated teeth and the history of dental examinations and treatment over a longer period in the future. Pregnant women and nursing mothers required an appropriate amount and balance of food for the development of their children, and it is important to educate them on the importance of oral hygiene as a supportive measure in this regard. Additionally, women should be educated on ways to reduce the number of unprotected teeth during pregnancy. Considering the lack of correlation between the number of untreated teeth and dental checkups together with the association between the frequency of toothbrushing and the number of untreated teeth, educating women with a high number of untreated teeth regarding the required frequency of toothbrushing may be meaningful. Furthermore, educating women on the oral environment and toothbrush instructions before pregnancy is important.

Gingival Bleeding

We found gingival bleeding in 646 of 907 women (71.2%): 199 (71.3%) in their teens and 20s, 408 (70.2%) in their 30s, and 39 (83.0%) in their 40s. Moreover, the number of healthy teeth in women in these age groups was 80 (28.7%), 173 (29.8%), and 8 (17.0%), respectively. No significant differences were observed in distribution. Only moderate correlations were found between gingival bleeding and periodontal pocket status (r=0.332), oral cleaning status (r=-0.381), and tartar buildup (r=0.290). However, the correlation coefficient between the frequency of toothbrushing and periodontal pockets was -0.053, indicating that gingival bleeding cannot be reduced by increasing the brushing frequency alone. Therefore, not only the frequency but also the toothbrushing technique is important. Therefore, guidance should be provided on proper toothbrushing techniques to prevent periodontal disease during pregnancy.

Limitations and future directions

One limitation of this study was that the pregnancy outcomes of the participants in the study were not recorded, making it impossible to analyze any potential associations. Additionally, recall bias in self-reported data and potential selection bias in the sample should also be considered. Future longitudinal studies should investigate the impact of oral health on pregnancy, postpartum health, and lifelong dental health. 

## Conclusions

In conclusion, within the limitation of this study, brushing teeth three times daily, including during pregnancy, is ideal for maintaining oral health. However, the correlation between the number of untreated teeth and toothbrushing frequency was low in this study. This study underscores the need for obstetricians and dentists to recognize the role of oral hygiene management in pregnancy and to advocate for regular dental checkups. By fostering awareness and encouraging preventive care, valuable opportunities are created for improving overall maternal health. Additionally, further research on the association between dental health and obstetric complications in Japan holds promise for contributing to the reduction of pregnancy-related complications.
